# Management of Osteomyelitis in Autosomal Dominant Osteopetrosis: A Rare Case Report

**DOI:** 10.7759/cureus.62660

**Published:** 2024-06-19

**Authors:** Mishal Almutairi, Ahmed Alharbi, Horiyah Almutairi, Mohamed F Shemis, Masaad S Almutairi, Faris Almutairi

**Affiliations:** 1 General Dentistry, Qassim University, Buraydah, SAU; 2 Oral and Maxillofacial Surgery, Qassim University, Buraydah, SAU; 3 Pharmacy Practice, Qassim University, Buraydah, SAU

**Keywords:** mandible, albers-schönberg disease, osteopetrosis, autosomal dominant, osteomyelitis

## Abstract

Albers-Schönberg disease, also known as osteopetrosis or marble bone disease, is a rare genetic disorder characterised by increased cortical bone mass due to dysfunctional osteoclast cells. This case report presents a 34-year-old male with autosomal dominant osteopetrosis (ADO), who was referred for evaluation and treatment of a chronic mandibular abscess with associated osteomyelitis and fistula. The patient's medical history included multiple fractures necessitating open reduction and internal fixation. Radiological examinations revealed the presence of chronic osteomyelitis in the mandible, marked by an increase in bone density and obliteration of medullary spaces. The treatment approach included surgical debridement, extraction of adjacent teeth, sequestrectomy, and antibiotic therapy. Notably, Enterobacter cloacae bacteria were identified through culture, leading to a tailored antibiotic regimen. Follow-up assessments, including clinical photographs and postoperative CT scans, were conducted to monitor the patient's progress. Histopathological examination confirmed osteomyelitis showing both viable and non-viable bone, surrounded by significant inflammatory infiltrate. This case underscores the complexity of managing osteomyelitis in patients with osteopetrosis and highlights the importance of early diagnosis, particularly before dental extractions, to prevent disease exacerbation. The rarity of this condition emphasises the need for further research and awareness among healthcare providers for optimal patient care.

## Introduction

Albers-Schönberg disease, also known as osteopetrosis or marble bone disease, was first reported by German radiologist Albers-Schönberg in 1904 [1]. This rare genetic disorder is characterised by increased cortical bone mass due to dysfunctional osteoclast cells, which, although present in normal quantities, tend to be inactive or inefficient [2]. There are two primary genetic forms of osteopetrosis: Autosomal Recessive Osteopetrosis (ARO) and Autosomal Dominant Osteopetrosis (ADO) [3].

ARO is marked by dense, brittle bones, anaemia, and nerve compression, severely impacting patients' quality of life and often leading to early death. Conversely, ADO, the more common variant with an incidence of 1 in 20,000, typically remains dormant in youth [4]. Its clinical presentation includes short stature and robust bones [5]. Diagnosis is generally through radiographic and clinical examination, with patients experiencing bone pain, frequent fractures, and infections following dental procedures [6].

One major complication in ADO is osteomyelitis following tooth extraction, affecting about 10% of patients [7]. This complication arises from microbial adherence at the extraction site and reduced vascularization, leading to symptoms like persistent bone exposure and necrotic bone sequestration [8]. Management of osteomyelitis in osteopetrosis patients involves surgical interventions and antibiotic therapy [9]. Given the rarity of this condition, this paper intends to present a case report of osteopetrosis in a 34-year-old male patient.

## Case presentation

**Figure 1:**
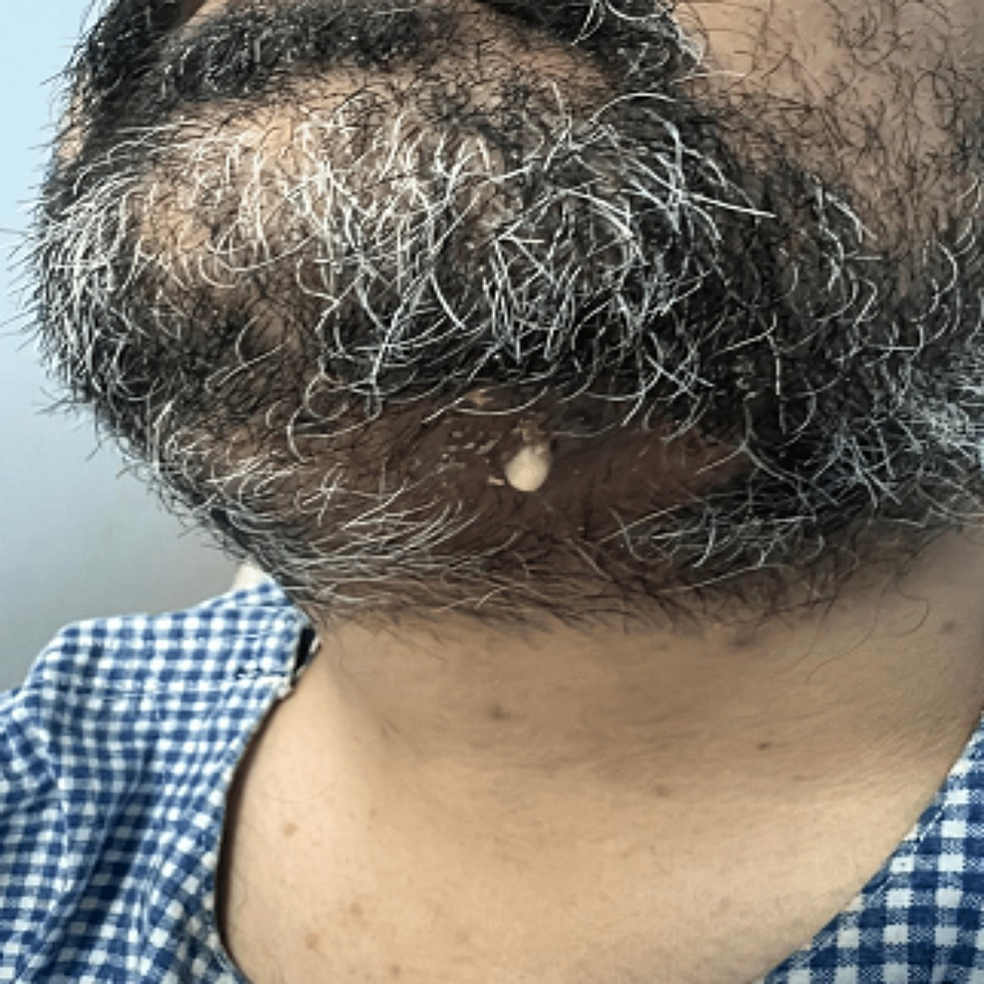
Chronic Mandibular Osteomyelitis with Fistula Formation in Autosomal Dominant Osteopetrosis

**Figure 2:**
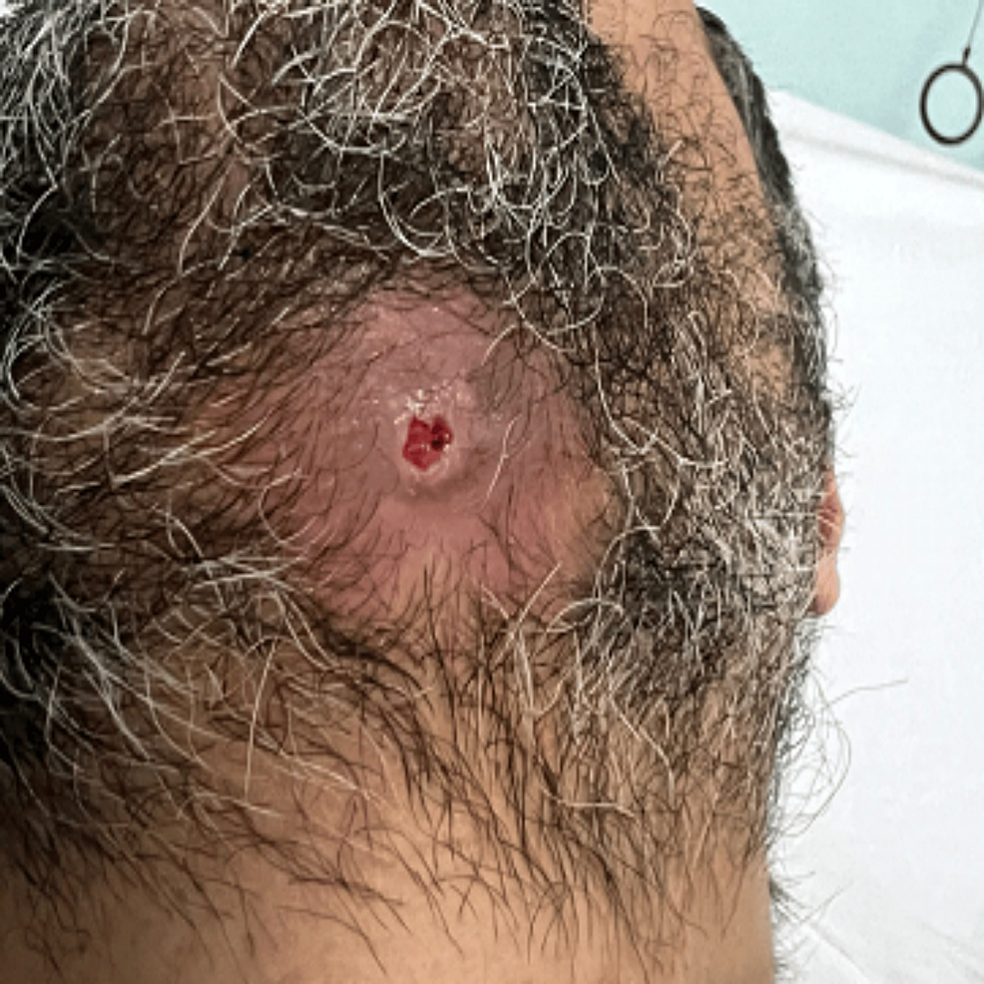
Postoperative Clinical Image of Cutaneous Fistula in Osteopetrosis-Related Osteomyelitis

**Figure 3:**
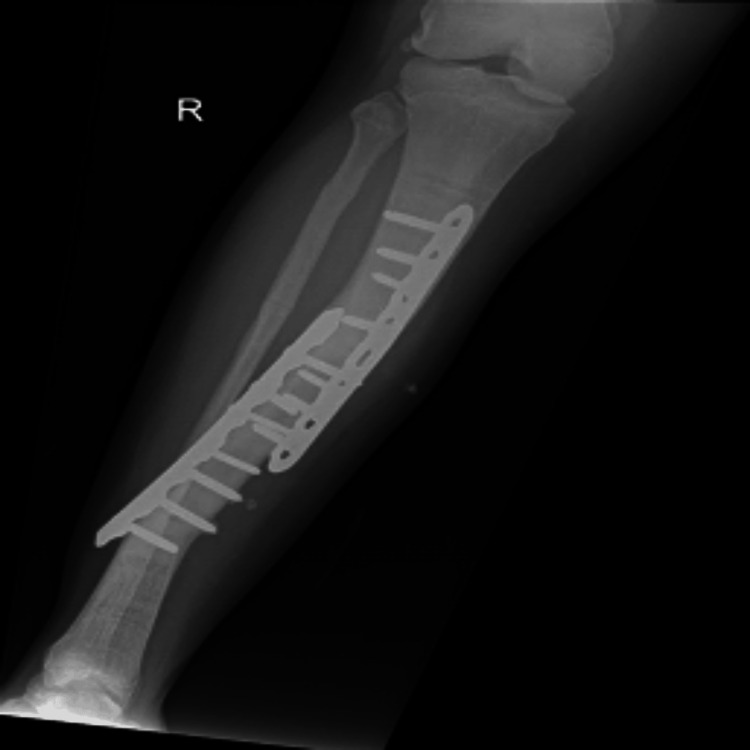
Radiographic Evidence of Surgical Fixation in Osteopetrosis-Related Fracture Management

A 34-year-old Caucasian male was referred from the Emergency Department at Buraidah Central Hospital in the Qassim Province of Saudi Arabia to the Oral and Maxillofacial department for assessment and treatment of a long-standing abscess with mild pain, swelling, and a fistula on the left side of his jaw. On external examination, the patient exhibited swelling under the left side of the jaw and a pus-discharging fistula on the left side of the neck (Figure 1). The swelling had been present for one month, while the fistula had been draining for a week before the referral (Figure 2). During the intraoral examination, heavy calculus deposits were observed, along with several loose teeth and swelling in the left mandibular vestibule. The patient's medical history revealed a diagnosis of autosomal dominant osteopetrosis, with multiple previous fractures that required open reduction and internal fixation (ORIF), including the right femur, right tibia left tibia, and both bones of the right and left forearm (Figure 3).

Management

The treatment plan included intravenous antibiotic therapy, removal of necrotic bone, sequestrectomy, extraction of adjacent teeth, and saucerization of the bone. Subsequently, the patient was admitted to the surgical ward for intravenous antibiotics and close monitoring. Initial antibiotic therapy included Cefazolin 1 gm, Metronidazole 500 mg, and clindamycin 600 mg for the first two weeks. Following the identification of Enterobacter cloacae bacteria in culture from the persistent infection, the antibiotic treatment was adjusted to ertapenem based on the culture results. Multiple extraoral clinical photographs were taken at different time points to assess the resolution of the fistula.

Radiological examination

**Figure 4:**
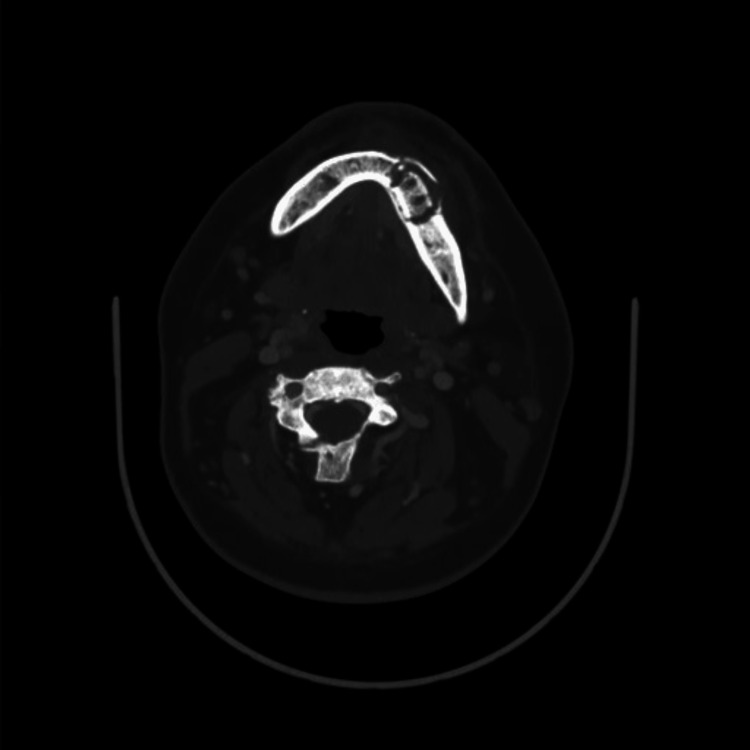
Axial CT Scan Depicting Mandibular Osteomyelitis in Osteopetrosis

A coronal CT scan revealed an ill-defined radiolucent area surrounding a radiopaque sequestrum in the posterior left side of the mandible, indicating chronic osteomyelitis. The scan also showed an increase in bone density, obliteration of medullary spaces, and a loss of differentiation between the cortex and medulla. Furthermore, the horizontal section displayed extensive bone resorption and dissolution of the buccal plate (Figure 4).

Histopathological examination

The excised sample comprised multiple firm, greyish-brown bone fragments, collectively measuring 2.5 by 1 centimetres, all enclosed within a single cassette. Microscopic examination revealed a mix of viable and non-viable bone tissue enveloped by acute and chronic inflammatory infiltrate, corroborating the clinical suspicion of osteomyelitis. The soft tissue surrounding the bone displayed severe inflammation, although the skin covering the specimen was noted to be unremarkable.

**Figure 5:**
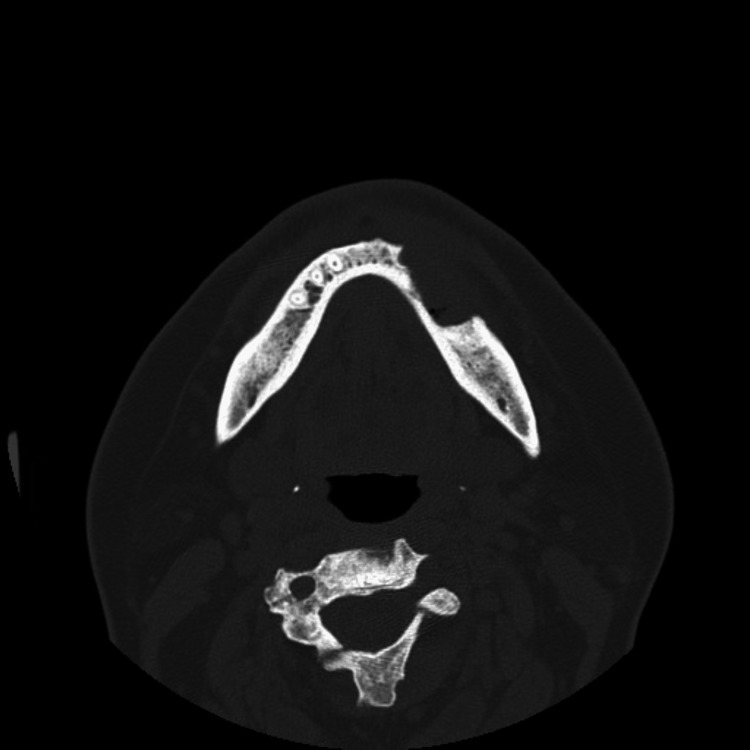
Post-Operative Axial CT Scan for the Surgical Site

**Figure 6:**
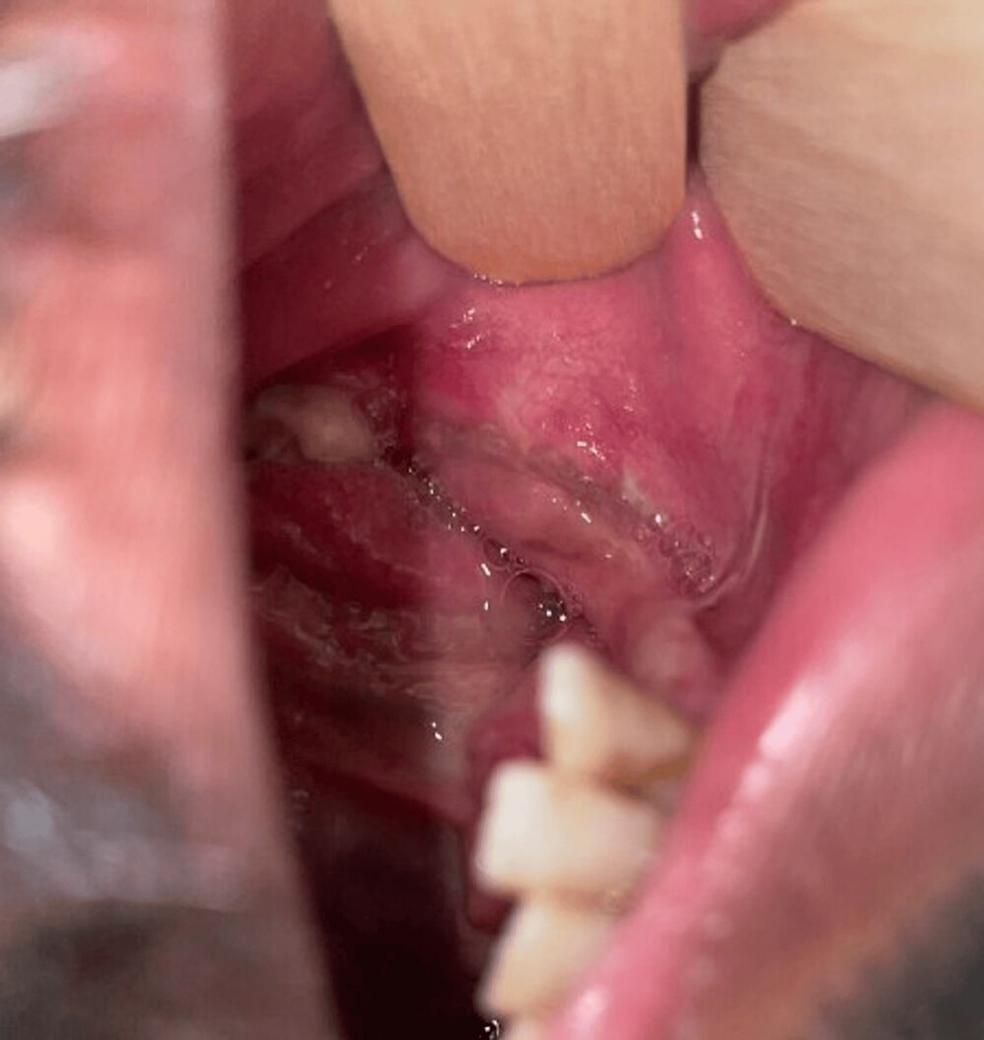
Intraoral View after Ten Months Follow-up

An additional CT scan was scheduled for evaluation eight weeks after the surgery (Figure 5). Ten months follow-up after the surgical intervention demonstrated an absence of the ongoing infections in the face and oral tissues (Figure 6).

## Discussion

This report discusses a case of autosomal dominant osteopetrosis (ADO) complicated by the development of osteomyelitis in the mandible. The onset of osteomyelitis and fistulas in this case was directly associated with facial trauma and tooth extractions. Initially, surgical saucerization and antibiotic treatment were employed to address the osteomyelitis in the mandible. However, due to persistent symptoms, partial resection of the affected area had to be considered.

Osteomyelitis is a known complication of osteopetrosis, occurring in approximately 10% of patients, typically affecting the jaw [10]. The development of osteomyelitis in these cases is primarily attributed to altered white blood cell function and reduced vascular supply [11]. Additionally, the diminished blood circulation limits the effectiveness of antibiotics at the infection sites [12]. In this particular case, the significant reduction in bone marrow cavities and periodontal ligament spaces undoubtedly contributed to impaired blood circulation, making the patient more susceptible to infections.

In contrast to the radiographic diagnosis of osteopetrosis, managing osteoporotic patients presents a complex challenge for physicians [13]. The treatment approach may encompass surgical drainage, antibiotic therapy, sequestrectomy, tooth extraction, saucerization, decortication, bone resection, and hyperbaric oxygen therapy [14]. However, only bone resections and hyperbaric oxygen therapy have been clinically validated as effective treatments for osteomyelitis in osteoporotic patients [15].

In conclusion, this case underscores the importance of managing osteomyelitis secondary to osteopetrosis and emphasizes the need for early diagnosis in patients facing dental extractions, particularly when extracting a mandibular tooth, to prevent exacerbation of the condition.

## Conclusions

This case report sheds light on the challenging management of osteomyelitis in a patient with autosomal dominant osteopetrosis. The rarity of this genetic disorder, coupled with the complexity of its clinical manifestations, underscores the need for early diagnosis and specialized care. Successful management involves a multidisciplinary approach, including surgical intervention, tailored antibiotic therapy, and close monitoring. Furthermore, this case highlights the importance of preoperative evaluation and caution when considering dental extractions in patients with ADO to prevent the development or exacerbation of osteomyelitis. Continued research and awareness are vital to improving the quality of life for individuals with this rare condition and enhancing healthcare providers' ability to provide effective treatment.
